# *Programmed Death Ligand 1*; An Immunotarget for Renal Cell Carcinoma

**DOI:** 10.31557/APJCP.2019.20.10.2951

**Published:** 2019

**Authors:** Deepika Chandrasekaran, Sandhya Sundaram, Kadhiresan N, Padmavathi R

**Affiliations:** 1 *Department of Physiology, *; 2 *Department of Pathology,*; 3 *Apollo Speciality Hospital, Teynampet,*; 4 *Department of Physiology, SRMC and RI, Porur, Chennai, India. *

**Keywords:** Renal cell cancer, PD-L1, prognosis, immunotherapy, immunohistochemistry

## Abstract

**Background:**

In this era of developing targeted therapies and immunotherapies as a treatment for renal cell carcinoma (RCC), *Programmed death ligand 1* (*PDL1*) as a novel biomarker for RCC is analysed in our study. About 90% of all renal cancers are Renal Cell Carcinoma. Most cases are diagnosed incidentally. 17% of cases are advanced at the time of diagnosis. *PDL1* being a trans-membrane cell surface protein is expressed on the tumor cells and is found to have a chief role to inhibit the T cell immune response. It is essential to improve the host immunity by targeting the *PD1/PDL1* pathway, thereby destroying the tumor progression. **Aim: **The aim of this study was to evaluate the expression of *PDL1* in tumor cells and adjacent normal tissue among the renal cell carcinoma patients and assess the relation between the *PDL1* expression and the tumor characters.

**Methods:**

This is a retrospective study. Ethical clearance was obtained from the institution. 150 histopathologically proven RCC cases were chosen. Immunohistochemistry using a *PD-L1* rabbit monoclonal antibody was performed on paraffin embedded formalin fixed tissue blocks. Q scoring was done to calculate the expression of *PDL1*.

**Statistical analysis:**

Chi square test was done to assess the comparison between the *PDL1* expression in tumor cells and their characteristic features like histology, grade and stage. SPSS (version 20.0) was used for analysis. P value <0.05 was considered significant. It also explains the heterogenous nature of* PDL1* as it expressed more in the aggressive pathologic characters like high grade. **Results: **Positive PD-L1 expression was seen in 44% of tumors. Significant association was observed between high WWHO ISUP grading and positive *PDL1* expression (p=0.028). It was expressed in 75% of the sarcomatous type of RCC and 46.8% of clear cell RCCs. **Conclusion: **Our study suggests that blocking *PD1/PDL1* pathway may become an effective mode of treatment in cancer immunotherapy especially for Renal Cell Carcinomas. Our findings confirmed the significant association between expression of *PDL1 *and the high graded tumors which proves it to be an important prognostic factor.

## Introduction

In the recent years with the advancement in the knowledge about the disease biology and the introduction of targeted agents and immunotherapies, a significant progress in the treatment of renal cell carcinoma(RCC) is noted. Renal cell carcinoma (RCC) is the sixth most frequently diagnosed cancer in men and tenth in women (Siegel et al., 2018). It is a very aggressive malignancy. About 90% of all renal cancers are Renal Cell Carcinoma. Most cases are diagnosed incidentally. At the time of diagnosis, many cases are small tumors and can be surgically removed whereas 17% of cases are advanced (Capitanio and Montorsi, 2016). Around 30% of patients will present metastatic disease at the time of diagnosis and metastases are found in 30% to 40% of those initially treated in curative intent RCC ranks as the 13^th^ common cause of death worldwide (Hutson, 2011). Inspite of the advancements made in the diagnosis, treatment strategies of RCC; it is observed in the last two decades that RCC has become a lethal urological malignancy (Chow and Devessa, 2008). High incidence is seen in the western population especially in North America followed by Western Europe. Asian countries have an age standardised rate of 0.6% in western Asia and 0.3% in eastern Asia. According to the GLOBOCAN 2018, the age standardised rate of kidney cancers incidence in USA is 10 per 1,00,000/year and mortality around 2.3 per 100,000/year. In European countries, the incidence is around 9 per 100,000/year and mortality 3 per 100,000/year. In the last few decades the incidence of kidney cancers in India is around 1 per 1,00,000/year and mortality 0.8 per 100,000/year. It is now increasing at a faster rate in the Indian population (Ferlay et al., 2015). RCC is resistant to both chemotherapy and radiotherapy. Nephrectomy is considered the treatment of choice for most RCCs (Rossi et al., 2018). 

**Table 1:** Baseline Parameters of the Study

Characteristics	No. of patients (n)	%
Gender		
Male	111	74
Female	39	26
Histology: clear cell		
clear cell	109	72.7
Papillary	14	9.3
Chromophobe	22	14.7
Collecting duct	1	0.7
Sarcomatous	4	2.7
Grade		
I	23	15.3
II	94	62.7
III	19	12.7
IV	14	9.3
Stage	63	42
I		
II	43	28.7
III	28	18.7
IV	16	10.7
*PDL1 *positive	66	44
*PDL1* negative	84	56
	Median	Min to Max
Mean Age	55	23 to 90
Mean tumor size	7.3	1.8 to 21

**Table 2:** Comparison of the PDL1expression with the Clinical and Pathologic Features of Renal Cell Carcinoma Patients

Feature	PDL1 + (66)	PDL1 – (84)	P value
Age at surgery			
<= 50	15 (31.9%)	32 (68.1%)	
>50	51 (49.5%)	52 (50.5%)	0.04
Sex			
Male	54 (48.6%)	57 (51.4%)	0.053
Female	12 (30.8%)	27 (69.2%)	
Sub-types			
Clear cell	52 (46.8%)	58 (53.2%)	
Papillary	4 (28.6%)	10 (71.4%)	
Chromophobe	7 (31.8%)	15 (68.2%)	
Sarcomatoid	3 (75%)	1 (25%)	0.21
Collecting duct	0 (0%)	1 (100%)	
Grade			
I	10 (43.5%)	13 (56.5%)	
II	35 (37.2%)	59 (62.8%)	
III	10 (52.6%)	9 (47.4%)	0.028
IV	11 (78.6%)	3 (21.4%)	
Stage			
I	28 (44.4%)	35 (55.6%)	
II	15 (34.9%)	28 (65.1%)	
III	14 (50.0%)	14 (50%)	0.417
IV	9 (56.3%)	7 (43.8%)	

Statistical analysis was done using Chi square test to compare the *PDL1 *expression and tumor characteristics; p value <0.05 was considered as significant

**Figure 1:**
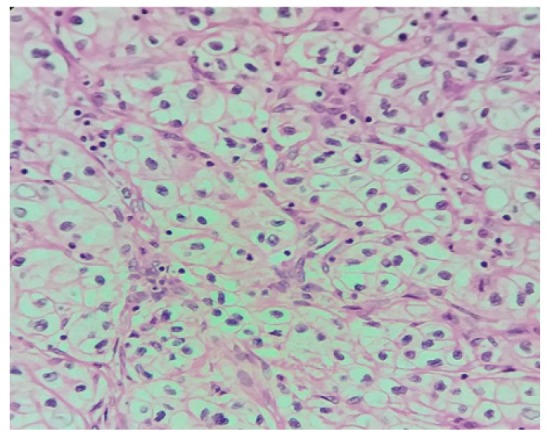
H and E Clear Cell Renal Cell Carcinoma

**Figure 2:**
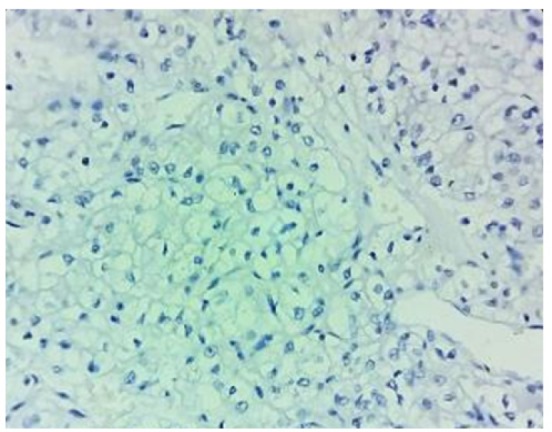
*PDL1* Negative Score: 0

**Figure 3:**
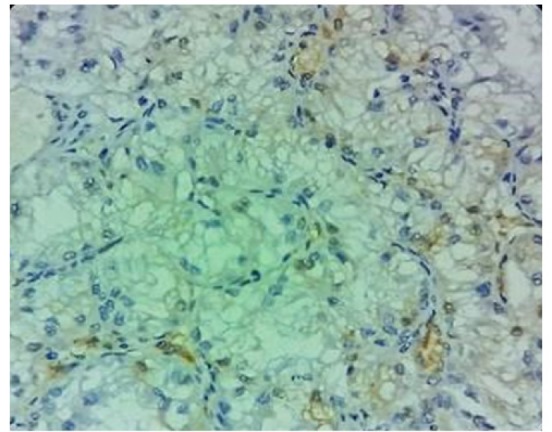
*PDL1* Expression Score: 1

**Figure 4:**
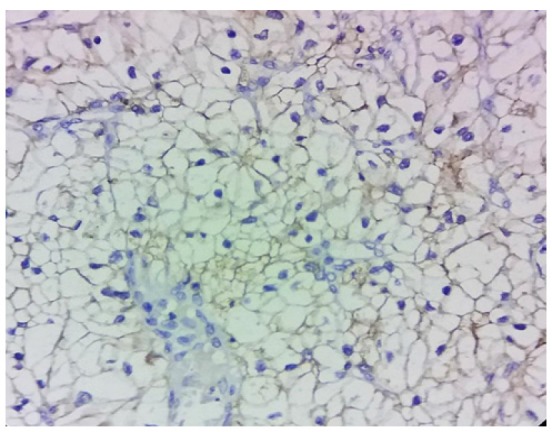
*PDL1* Expression Score: 2

**Figure 5:**
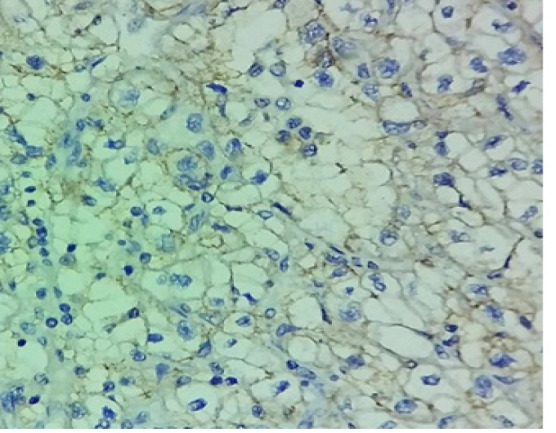
*PDL1* Expression Score: 3

**Graph 1:**
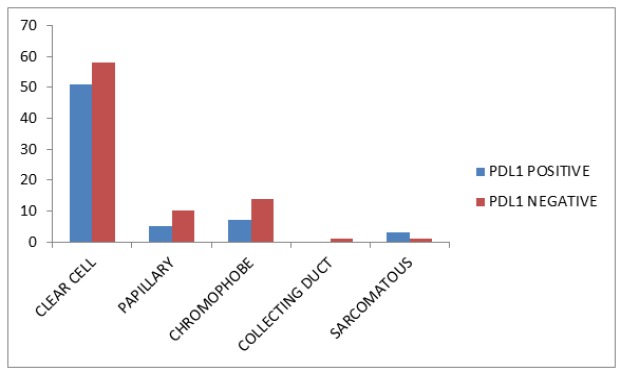
Distribution of *PDL1* Expression among the Tumor Histological Subtypes

**Graph 2:**
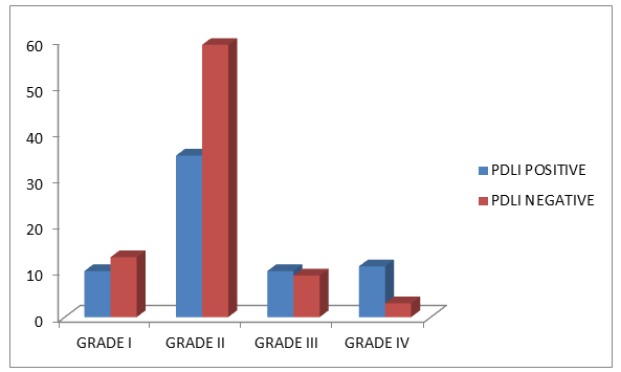
Distribution of* PDL1* Expressions among the Furhman Nuclear Grades

A number of clinical trials have proved the effectiveness of targeted therapies in RCC. VEGF and mTor pathway inhibitors play a major role in designing the treatment strategy. They inhibited the angiogenic activity of the tumor and arrested its further growth. But all these fail in the case of metastatic RCC (Choueiri et al., 2015). Hence other modalities of treatment had to be tried. Immunotherapy like interferon –α and interleukin 2 have been found to improve the survival rates in a very small percentage of the patients (Fyfe et al., 1995). 

*Programmed death ligand 1* which is a trans-membrane cell surface protein seen expressed on the tumor cells. Their chief role is to inhibit the T cell immune response. It binds with* PD1*which is a T cell surface protein and inactivates it, thereby promoting tumor growth and metastasis (Harshman et al., 2014). Thus it is essential to improve the host immunity by targeting the *PD1/PDL1 *pathway. These blocks the* PD1* and* PDL1* binding, thus the inactivated T cell becomes active again and destroys the cancer cell (Gong et al., 2018). Before performing this immunotargeted therapy, we should select the patients who respond to this type of targeted immune therapy. For this we need to study the expression of *PDL1* in the tumor cells of the RCC patients by Immunohistochemistry.

*Aim*

The aim of this study was to evaluate the expression of PDL1 in tumor cells and adjacent normal tissue among the renal cell carcinoma patients and assess the relation between the *PDL1* expression and the tumor characters

## Materials and Methods

This is a cross sectional study. Ethical clearance and the needed permissions were obtained from Sri Ramachandra Institute of Higher Education and Research. All Renal cell carcinoma patients who underwent nephrectomy in Sri Ramachandra Hospital during the period January 2010 to September 2018 were initially chosen for the study. Among them 150 histo pathologically proven RCC cases were shortlisted. Their corresponding paraffin embedded formalin fixed blocks were carefully chosen. Their relative case reports were analysed from the medical records department. There were 111 males and 39 females included in the study. The mean age is 55 and the mean tumor size is 7.3cm. Most of the cases were admitted with the complaint of abdominal pain (73) and hematuria (39). Furhmans nuclear grading was done to assess the grade of the tumor. TNM staging was done to stage the tumor. 

Immunohistochemistry (Biotin Streptavidin Immunoperoxidase method) was done to assess the expression of PDL1 in the tumor cells. The tissue blocks were first sliced 4um thick tissue section and fixed to a slide followed by dewaxing and hydration. Then hydrogen peroxide was added and rinsed in PBS. Then citrate buffer was added, cooled and rinsed with PBS. Later* PDL1* rabbit monoclonal antibody (E1L3N; cell signalling technology, 1:25 dilution) was used and incubated overnight at 4ºC. Again it was rinsed with PBS and the universal DAB dilution kit was used to visualize the primary antibody (Cogswell et al., 2017). Slide was then counterstained with hematoxylin, dehydrated with alcohol and sealed. The prepared slide was focussed under the microscope at high magnification x200. Brown colouration was noticed in the cell membrane of the tumor cells which explains the expression of *PDL1* in the tumor cells (Choueiri et al., 2015. Then Q scoring was done to calculate the expression of *PDL1*. Q = P X I; P – percentage of cells showing *PDL1* expression; I – intensity of the stain taken up by the cells (Namnak et al., 2015). 

The percentages of* PDL1* expression were assessed according to the extent of staining. Figure 1 shows the hematoxylin and eosin stained clear cell RCC slide. Negatively stained slides or showing expression in tumor cells < 5% was scored 0 (Figure 2), weakly expressed slides or tumor cells >5% were scored 1 (Fgure 3). Moderate expressed slides and cells >5% were scored 2 (Figure 4) and strongly expressed in cells > 5% were scored 3(Figure 5). The samples were subsequently subdivided into *PDL1* positive (scores 1, 2,3) and PDL1 negative (score 0) (Mahoney et al., 2015). The arrow marks shown in the figures 3, 4 & 5 show the membrane staining of* PDL1* in the clear cell types of RCC.

*Statistical Analysis*

Chi-squared tests were used to compare the PDL1 expression with the tumor characteristics. All analyses were conducted in SPSS (version 20.0). All P-values were two-sided and those <0.05 were considered statistically significant.

## Results

The characteristics the subjects involved in the study are highlighted in the Table 1. The study included 150 subjects with RCC. According to the WHO-ISUP grading, the tumors were grouped as high grade tumors (III, IV) and low grade tumors (I, II). Among the 150 samples, 66 (44%) expressed PDL1 expression in the tumor cell surface whereas 84 were negatively stained. The classical histological subtypes of RCC are clear cell (ccRCC), papillary, chrompphobe, collecting duct and sarcomatous. Graph 1 explains the distribution of *PDL1* expressing tumors belonged to the sarcomatous subtype (75%) which is mostly high grade and high staged tumors. Graph 2 explains the increased expression of *PDL1* among the high grade tumors. From the Table 2, it clearly shows us that *PDL1* expression is significantly associated with the WHO ISUP grading (0.028). On the other hand,* PDL1* was not associated with age, gender, stage and histology. 

## Discussion

Systemic therapies targeting vascular endothelial growth factor (VEGF) and mammalian target of rapamycin (mTOR) pathway form the major advances in treatment of metastatic RCC. In the later decades their effectiveness had no much role in metastatic cases (Hutson et al., 2011). Many promising results were obtained recently after the entry of the new modality of treatment – the *PD1/PDL1* pathway inhibition. *PDL1* is being studied as the biomarker for tumors like melanoma, RCC and NSCLC (Thompson et al., 2006). It is found to inhibit the *PD1/PDL1* pathway thereby arresting the tumor progression. But it is still not a well-established biomarker due to the lack of researches in this field of immunotherapy. To date many clinical trials are going on to show the positive responses of* PDL1*.

According to the studies from the western population, Renal cell carcinoma (RCC) represents 2% to 3% of all cancers, corresponding to 338,000 new cases diagnosed each year worldwide. If the incidence and mortality tend to decrease in Western Europe, recent data from the United States show a continued increase in incidence. The expression of *PDL1* has been studied in various American and European countries. But very few studies have been done in India in the renal carcinoma cases. In the last few decades, due to the increase in incidence of RCC in the Indian population, many research works nave begun (Jacquet et al., 2019).

Our study showed 44% of the renal cell carcinoma tissues expressed positive *PDL1* in the tumor cell membrane. It was expressed in 75% of the sarcomatous type of RCC and 46.8% of clear cell RCCs and a significant association was between the *PDL1* expression and the high graded tumors. Dong et al., (1999) described the Tcell surface protein *PDL1* expressed by the tumor cells. These tumor cells expressing *PDL1* have been found to inhibit the T cell mediated immunity and as a result help in the progression of the tumor (Dong et al., 1999). There are a few literatures showing the relation between the *PDL1* expression and the improvement in the prognosis of the RCC patient. Thompson et al was the first to do a detailed study about *PDL1* among the RCC patients in 2004 (Thompsonet al., 2004). Many researchers studied the relation between PDL1 expression and poor prognosis. Some studies showed poor survival among the metastatic patients expressing PDL1 (Rini et al., 2006). Interestingly few literatures showed no correlation in the* PDL1* expression between the primary tumors and metastatic tumors.

In breast cancer, *PDL1* was associated with poor prognosis. In gastric cancers, *PDL1* positivity was associated with high staged tumors and overall survival (Iwai Y, 2002). A review study in non-small cell lung carcinoma specified the association of *PDL1* expression with tumor differentiation as well as overall survival rate. These NSCLC patients were found to get benefitted with *PD1/PDL1* inhibitor – Nivolumab. Pembrolizumab has also been studied in NSCLC cases (Yamaguchi et al., 2017). There are few studies which showed *PDL1 *negative tumors responded well to the nivolumab than the *PDL1 *expressing tumors. Many combination studies are also going on where anti VEGF therapy and anti* PDL1 *therapy for metastatic cases showed reasonable benefits in clear cell RCC. *PDL1* levels in serum samples were studied in few ccRCC patients but failed to prove and significance (Kruger et al., 2017). 

In our study we studied the weak, moderate and strong expression of *PDL1* in different subtypes of RCC. Their association with the high graded tumor gives the hope to proceed with further research in this field to prove the potential role of *PDL1* among the RCC tumors. According to Joseph et al. sarcomatous type of RCC expressed stronger *PD-L1* than RCC without sarcomatous features which substantiates our study which also showed the greater expression of *PDL1* among the sarcomatous RCCs (Joseph et al., 2015).

Kammerer-Jacquet et al demonstrated that the *PD-L1* expression was associated with noninactivated VHL tumors. Few molecular studies have also showed that strong *PDL1* expression was seen in mutated tumors and weak expression among the wild type tumors (Messai et al., 2016). Some studies have been done in the VHL mutated ccRCC subjects. They showed the efficacy of *PDL1* inhibitors in these subjects was due to the hypoxia inducible factor (HIFα) accumulation which is the effect of VHL inactivation. These reports suggest that *PDL1* may be a favourable predictive biomarker for VHL mutated cases, metastatic RCCs, poor prognostic cases and targeted therapy failed cases (Rini et al., 2006). 

In our study we did find that *PD-L1* was differentially expressed in the same tumor-the higher the nuclear grade was, the more evident expression of *PD-L1* was observed, suggesting that* PD-L1* expression was possibly required to be evaluated in metastases in order to more accurately predict the therapeutic effect of immune checkpoint inhibitors (Topalian et al., 2012). It also explains the heterogenous nature of PDL1 as it expressed more in the aggressive pathologic characters like high grade. This gives us a valuable point that *PDL1* can become a potential biomarker. At present, the clinical evaluation of tumor heterogeneity is a promising issue to improve clinical oncology. This intra-tumor heterogeneity (ITH) is very much related to cancer progression, resistance to therapy, and recurrences. This peculiar nature of the tumour is different for different patients (Stant et al., 2018).

The clonal ITH indicates the genomic instability that influences aggressiveness and treatment. The nonclonal ITH is functional, microenvironment related. Both the types influence each other. Intra-tumor heterogeneity analysis is the strategic method for identifying efficient treatments modalities. Every aspect of ITH should be considered as a mojor source of research information. Together with preclinical conditions of the biological materials and standardization of the analytical methods, the best treatment strategy can be designed for all RCCs. This ITH assessing genomic engineering method has already been used to study mechanisms of lung cancer cell resistance to EGFR inhibitors and to investigate on combined drug therapies (Beksac et al., 2017).

Research should be done analysing the association between the various risk factors of RCC and their immunhistochemical findings. It helps us to choose which patient might respond to which targeted therapy. Also assessing the heterogenous nature may bring about a revolution in the field of RCC treatment management. But it’s a vast field and needs a tremendous literature research before beginning such a correlation study which is our next field of interest.

One major limitation in this study was the lack of proper standardised immunohistochemical techniques for the assessment of *PDL1*. The difference in the staining methodologies as well as the usage of different antibodies for staining the tumor cells in various studies also prevented the assessment of their performance. Hence standardizing the staining procedure, choosing the antibody and the scoring technique is necessary before using* PD-L1* as a predictive biomarker.

In conclusion, In this era of evolving targeted therapies and immunotargeted therapies for RCC, *PDL1* as a novel biomarker was analysed in this study. The association of *PDL1 *with high graded RCCs was statistically significant. This suggests that blocking *PD1/PDL1* pathway may be effective method in cancer immunotherapy. Hence targeted therapies for *PDL1* like Nivolumab can be of potential therapeutic benefits in future. Further studies are needed in this field to prove that* PDL1* can become an independent marker for prognosis of RCC.

## References

[B1] Abraham GP, Cherian T, Mahadevan P (2016). Detailed study of survival of patients with renal cell carcinoma in India. Indian J Cancer.

[B2] Beksac AT, Paulucci DJ, Blum KA (2017). Heterogenecity in renal cell carcinoma. Urol Oncol.

[B3] Brahmer JR, Tykodi SS, Chow LQ 2012) Safety and activity of anti-pd-l1 antibody in patients with advanced cancer. N Engl J Med.

[B4] Callea M, Albiges L, Gupta M (2015). Differential expression of PDL1 between primary and metastatic sites in clear cell renal cell carcinoma. Cancer Immunol Res.

[B5] Camp RL, Charette LA, Rimm DL (2008). Validation of tissue microarray technology in breast carcinoma. Lab Invest.

[B6] Capitanio U, Montorsi F (2016). Renal cancer. Lancet.

[B7] Choueiri TK, Fay AP, Gray KP (2014). PD-L1 expression in nonclear-cell renal cell carcinoma. Ann Oncol.

[B8] Choueiri TK, Figueroa DJ, Fay AP (2015). Correlation of PD-L1 tumor expression and treatment outcomes in patients with renal cell carcinoma receiving sunitinib or pazopanib: Results from COMPARZ, a randomized controlled trial. Clin Cancer Res.

[B9] Chow WH, Devessa SS (2008). Contemporary epidemiology of renal cell cancer. Cancer J.

[B10] Cogswell J, Inzunza HD, Wu Q (2017). An analytical comparison of dako 28-8 PharmDx assay and an E1L3N laboratory-developed test in the immunohistochemical detection of programmed death-Ligand 1. Mol Diagn Ther.

[B11] Dall’Oglio MF1, Ribeiro-Filho LA, Antunes AA (2007). Microvascular tumor invasion, tumor size and fuhrman grade: a pathological triad for prognostic evaluation of renal cell carcinoma. J Urol.

[B12] Delahunt B, Cheville JC, Martignoni G (2013). Members of the ISUP renal tumor panel The International Society of Urological Pathology (ISUP) grading system for renal cell carcinoma and other prognostic parameters. Am J Surg Pathol.

[B13] Dong H, Zhu G, Tamada K, Chen L (1999). B7-h1, a third member of the b7 family, co-stimulates t-cell proliferation and interleukin-10 secretion. Nat Med.

[B14] Drake CG, Jaffee E, Pardoll DM (2006). Mechanisms of immune evasion by tumors. Adv Immunol.

[B15] Ferlay J, Soerjomataram I, Dikshit R (2015). Cancer incidence and mortality worldwide: sources, methods and major patterns in GLOBOCAN 2012. Int J Cancer.

[B16] Fyfe G, Fisher RI, Rosenberg SA (1995). Results of treatment of 255 patients with metastatic renal cell carcinoma who received high dose recombinant interleukin 2 therapy. J Clin Oncol.

[B17] Gong J, Raffle AC, Reddi S, Salgia R (2018). Development of PD-1 and PD-L1 inhibitors as a form of cancer immunotherapy: a comprehensive review of registration trials and future considerations. Cancer Immunol Immunother.

[B18] Gunturi A, McDermott DF (2014). Potential of new therapies like anti-pd1 in kidney cancer. Curr Treat Options Oncol.

[B19] Hanahan D, Weinberg RA (2011). Hallmarks of cancer: The next generation. Cell.

[B20] Harshman LC, Choueiri TK, Drake C, Stephen Hodi F (2014). Subverting the B7-H1/PD-1 Pathway in advanced melanoma and kidney cancer. Cancer J.

[B21] Hirai A, Yoneda K, Shimajiri S (2018). Prognostic impact of programmed death-ligand 1 expression in correlation with human leukocyte antigen class I expression status in stage I adenocarcinoma of the lung. J Thorac Cardiovasc Surg.

[B22] Hutson TE (2011). Targeted therapies for the treatment of metastatic renal cell carcinoma: Clinical evidence. Oncologist.

[B23] Iwai Y, Ishida M, Tanaka Y (2002). Involvement of pd-l1 on tumor cells in the escape from host immune system and tumor immunotherapy by pd-l1 blockade. Proc Natl Acad Sci U S A.

[B24] Jacquet SFK, Deleuze A, Saout J (2019). Targeting the PD-1/PD-L1 pathway in renal cell carcinoma. Inte J Mol Sci.

[B25] Jilaveanu LB, Shuch B, Zito CR (2014). PD-L1 expression in clear cell renal cell carcinoma: An analysis of nephrectomy and sites of metastases. J Cancer.

[B26] Joseph RW, Millis SZ, Carballido EM (2015). PD-1 and PD-L1 expression in renal cell carcinoma with sarcomatoid differentiation. Cancer Immunol Res.

[B27] Kruger S, Legenstein ML, Rösgen V (2017). Serum levels of soluble programmed death protein 1 (sPD-1) and soluble programmed death ligand 1 (sPD-L1) in advanced pancreatic cancer. Oncoimmunology.

[B28] Mahoney KM, Sun H, Liao X (2015). PD-L1 antibodies to its cytoplasmic domain most clearly delineate cell membranes in immunohistochemical staining of tumor cells. Cancer Immunol Res.

[B29] Messai Y, Gad S, Noman MZ (2016). Renal cell carcinoma programmed death-ligand 1, a new direct target of Hypoxia-inducible factor-2 Alpha, is regulated by von Hippel-Lindau gene mutation status. Eur Urol.

[B30] Moch H, Schraml P, Bubendorf L (1999). High-throughput tissue microarray analysis to evaluate genes uncovered by cDNA microarray screening in renal cell carcinoma. Am J Pathol.

[B31] Namnak S, Kittikowit W, Jutamas Wongphoom J (2013). The Role of immunohistochemistry in diagnosis of renal cell carcinoma subtypes. Asian Arch Pathol.

[B32] Pardoll DM. (2012). The blockade of immune checkpoints in cancer immunotherapy. Nat Rev Cancer.

[B33] Qing Y, Li Q, Ren T (2015). Upregulation of PD-L1 and APE1 is associated with tumorigenesis and poor prognosis of gastric cancer. Drug Des Devel Ther.

[B34] Rini BI, Jaeger E, Weinberg V (2006). Clinical response to therapy targeted at vascular endothelial growth factor in metastatic renal cell carcinoma: impact of patient characteristics and Von Hippel-Lindau gene status. BJU Int.

[B35] Rossi SH, Klatte T, Smith JU, Stewart GD (2018). Epidemiology and screening for renal cancer. World J Urol.

[B36] Sabatier R, Finetti P, Mamessier E (2015). Prognostic and predictive value of PDL1 expression in breast cancer. Oncotarget.

[B37] Shulyak A, Banyra O (2011). Radical or simple nephrectomy in localized renal Cell carcinoma: what is a choice?. Cent Eur J Urol.

[B38] Siegel RL, Miller KD, Jemal A (2018). Cancer statistics, 2018. CA Cancer J Clin.

[B39] Stanta G, Bonin S (2018). Overview on clinical relevance of intra tumoral heterogenecity. Front Med.

[B40] Tamura T, Ohira M, Tanaka H (2015). Programmed death-1 Ligand-1 (PDL1) expression is associated with the prognosis of patients with stage II/III gastric cancer. Anticancer Res.

[B41] Thompson RH, Dong H, Lohse CM (2007). PD-1 is expressed by tumor-infiltrating immune cells and is associated with poor outcome for patients with renal cell carcinoma. Clin Cancer Res.

[B42] Thompson RH, Gillett MD, Cheville JC 2004) Costimulatory B7-H1 in renal cell carcinoma patients: indicator of tumor aggressiveness and potential therapeutic target. Proc Natl Acad Sci U S A.

[B43] Thompson RH, Kuntz SM, Leibovich BC (2006). Tumor B7-H1 is associated with poor prognosis in renal cell carcinoma patients with long-term follow-up. Cancer Res.

[B44] Topalian SL, Hodi FS, Brahmer JR (2012). Safety, activity, and immune correlates of anti-pd-1 antibody in cancer. N Engl J Med.

[B45] Wang A, Wang HY, Liu Y (2015). The prognostic value of PD-L1 expression for non-small cell lung cancer patients: a metaanalysis. Eur J Surg Oncol.

[B46] Yamaguchi T, Sakurai K, Kuroda M, Imaizumi K, Hida T (2017). Different response to nivolumab in a patient with synchronous double primary carcinomas of hypo pharyngeal cancer and non-small-cell Lung cancer. Case Rep Oncol.

[B47] Zou W, Chen L (2008). Inhibitory b7-family molecules in the tumour microenvironment. Nat Rev Immunol.

